# First Isolation and Multilocus Sequence Typing of *Brucella canis* from a Subclinically Infected Pet Dog in China

**DOI:** 10.3390/vetsci9010022

**Published:** 2022-01-10

**Authors:** Guangwen Yan, Zidong Pang, Yan Hu, Ziyao Zhou, Haifeng Liu, Yan Luo, Zhihua Ren, Xiaoping Ma, Suizhong Cao, Liuhong Shen, Ya Wang, Liping Gou, Dongjie Cai, Yanqiu Zhu, Yalin Zhong, Wei Li, Xianpeng Shi, Guangneng Peng, Zhijun Zhong

**Affiliations:** 1 College of Animal Science, Xichang University, Xichang 615000, China; ygwdky@126.com; 2 Key Laboratory of Animal Disease and Human Health of Sichuan, College of Veterinary Medicine, Sichuan Agricultural University, Chengdu 611130, China; pzidong@163.com (Z.P.); hy837612495@163.com (Y.H.); zzhou@sicau.edu.cn (Z.Z.); 410140017@163.com (H.L.); lycjg@163.com (Y.L.); zhihua_ren@126.com (Z.R.); mxp886@sina.com.cn (X.M.); suizhongcao@126.com (S.C.); shenlh@sicau.edu.cn (L.S.); wangyayang@126.com (Y.W.); 72031@sicau.edu.cn (L.G.); dongjie_cai@sicau.edu.cn (D.C.); zhuyanqiu@sicau.edu.cn (Y.Z.); mxz723066@gmail.com (Y.Z.); liweisicau@163.com (W.L.); shixianpeng617@163.com (X.S.); pgn.sicau@163.com (G.P.)

**Keywords:** *B. canis*, pet dog, subclinical infection, public health, MLST

## Abstract

Canine brucellosis, a worldwide zoonotic disease, is mainly caused by *Brucella canis*. In the present study, we isolated a *Brucella* strain (CD3) from a subclinically infected pet dog in Sichuan Province, Southwestern China. Classical biotyping methods and molecular biological tests (BCSP31 and BcSS PCR) proved that the strain belonged to *B. canis*. Furthermore, *B. canis* CD3 and another two *B. canis* strains (WJ5 and YA4), which were all isolated from pet dogs in Sichuan, were genotyped using multilocus sequence typing (MLST). Our results showed that the three *B. canis* strains were identified as the same sequence type (ST21). The present study is the first to report *B. canis* strain from a subclinically infected pet dog in China, indicating a potential threat to public health posed by subclinical infections in pet dogs. We suggest that screening for *B. canis* should be incorporated into routine medical examination of pet dogs and other companion animals in areas with a history of animal or human brucellosis.

## 1. Introduction

Brucellosis, caused by Gram-negative, aerobic and facultative intracellular bacteria of the genus *Brucella*, is regarded as one of the most common zoonotic diseases worldwide [1,2]. *Brucella canis* is generally recognized as the pathogen of canine brucellosis, which primarily causes reproductive failure in dogs, including infertility and abortion [3]. *B. canis* infections in dogs have been increasing in China and other countries in recent years [3,4,5]. *B. canis* can also infect humans and cause severe diseases [5]. Human cases have been reported in many countries, such as China, the United States, Argentina, and Japan [6,7,8,9,10,11,12]. In some of these cases, pet dogs without clinical symptoms that had close contact with human brucellosis patients have been regarded as the source of infection [8]. As the number of pet dogs rises, the potential risk of human brucellosis due to the direct or indirect transfer from dogs to people is increased [13]. China is the most populous country in the world, and has raised about 50.08 million pet dogs as of 2018 [14]. The occurrence of *B. canis* infection in pet dogs in China was reported by our lab in 2015, in which the infected dog had clinical symptoms such as fever, enlarged lymph nodes and enlarged testicle [5]. However, subclinically infected pet dogs have not been reported in China.

Multilocus sequence typing (MLST) is a DNA sequence-based typing method that has been widely applied to microbial typing and epidemiological studies [15,16,17]. It was first used to genotype *Brucella* in 2007 and the result showed high differentiation among different species of *Brucella* strains [15]. Recent MLST studies on *Brucella* in China mainly focused on *B. abortus*, *B. melitensis*, and *B. suis*. The information of MLST of *B. canis* in China is relatively few. In 2019, Liu et al. had genotyped seven *B. canis* strains in Guangxi Province as ST21, enlarging our understanding of the genetic structure and the genetic diversity of *B. canis* population [18]. However, as far as we know, no report existed on MLST of *B. canis* in Sichuan Province.

In this study, bacteriological and molecular biological tests (BCSP31 and BcSS PCR) were used to identify a *B. canis* isolation (strain CD3) from a subclinically infected pet dog, and MLST method was then used to genotype the strain CD3 and another two *B. canis* strains (WJ5 and YA4), which were also isolated from pet dogs in Sichuan Province, China. For the first time, we isolated a *B. canis* strain from a subclinically infected pet dog in China, revealing a potential threat of subclinical infections in pet dogs to public health.

## 2. Materials and Methods

### 2.1. Clinical Sample

In August 2019, one male pet dog (golden retriever), aged three years and seven months, was tested for *Brucella* infection in Veterinary Medical Teaching Hospital of Sichuan Agricultural University at the request of the pregnant owner. Physical examination of the pet dog showed no clinical symptoms (medical history of reproductive ability was unknown). Blood samples were aseptically collected from a cephalic vein of the pet dog for serological tests and strain isolation.

In December 2015, one male dog (golden retriever), aged two years and five months, presented with low fever, enlarged lymph nodes, and unilateral testis. In October 2016, a male poodle, aged three years, presented with obviously enlarged testicle and undulating fever. Both pet dogs were diagnosed with canine brucellosis by Veterinary Medical Teaching Hospital of Sichuan Agricultural University. Two *B. canis* strains (WJ5, 2015 and YA4, 2016) were successfully isolated from the two cases and were stored in our lab [5].

### 2.2. Serological Tests

The Rose Bengal plate test (RBPT) and rapid slide agglutination test (RSAT) were performed with rough antigen to detect antibodies against *Brucella* in blood samples, as described previously [19].

### 2.3. Bacteriological Studies

Blood samples were plated and streaked on tryptic soy agar (Beijing Selarbio Science and Technology Co., Ltd., Beijing, China) for strain isolation. Plates were inoculated at 37 °C over 5 days [5]. Classical biotyping methods (including CO_2_ requirement, H_2_S production, hydrolysis of urea, agglutination in sera, growth on dyes and phage lysis tests) [20,21] were used to identify the isolated strain (named as CD3), and *B. canis* RM6/66 was used as a reference strain. All studies involving living bacteria isolation were performed under biosafety level 3 (BSL-3) laboratory (DYY-2019303075).

### 2.4. DNA Extraction

Before DNA extraction, specimens were boiled for 15 min. *Brucella* DNA was extracted using TIANamp Bacteria DNA extraction kit (TIANGEN Biotech Corporation, Beijing, China) according to the manufacturer’s instructions [5].

### 2.5. Polymerase Chain Reaction Tests

The isolated strain (CD3) was identified by two different PCR protocols using *Brucella* genus-specific (BCSP31) and *B. canis* species-specific (BcSS) primers, respectively. PCR amplification was conducted as described previously [13,22] and primers are listed in Table 1. *B. abortus* 544A, *B. melitensis* 16M, *B. suis* S2, and *B. canis* RM6/66 were used as reference strains. The PCR products (6 µL from each reaction mixture) were loaded for electrophoresis using a 1.5% agarose gel, after which the gel was stained with ethidium bromide and photographed [5].

### 2.6. MLST Genotyping

MLST genotyping was performed on the isolated *B. canis* strain (CD3). Two *B. canis* strains (WJ5 and YA4) from clinical cases of canine brucellosis in Sichuan were also analyzed [5]. Nine distinct genomic loci were analyzed, including seven housekeeping genes (*gap, aroA, glk, dnaK, gyrB, trpE, and cobQ*), one outer membrane protein (omp25), and one intergenic fragment (int_hyp) [15]. PCR amplification of the nine loci was performed, as described previously [15]. Primers are shown in Table 1. Sequences obtained from purified PCR products were aligned using MEGA X according to published MLST sequences in GenBank (accession numbers AM694191–AM695630) [17]. Distinct alleles identified at the nine selected loci were each given a numerical designation using a web-based MLST service (*Brucella* Base, https://pubmlst.org/Brucella, accessed on 9 August 2021) [17], and each unique allelic profile for the nine loci was identified as a sequence type (ST).

### 2.7. Analysis of MLST Data

Sequences of the nine loci were concatenated to produce a 4396 bp sequence for each genotype (ST). Sequence data of 3 *B. canis* strains (CD3, WJ5 and YA4) from pet dogs and 66 *Brucella* isolates in MLST database (https://pubmlst.org/Brucella, accessed on 9 August 2021), including four most common species (*B. abortus*, *B. melitensis*, *B. suis*, and *B. canis*) and two emerging species from marine mammals (*B. ceti* and *B. pinnipedialis*), were chosen for phylogenetic analysis using MEGA X. Neighbor joining trees were constructed using the Jukes–Cantor model and the percentage bootstrap confidence levels of internal branches were calculated from 1000 resamplings of the original data [15].

## 3. Results

### 3.1. Serological and Bacteriological Tests

RBPT and RSAT assays showed positive results for the tested blood samples. A suspected Brucella strain (CD3) was successfully isolated from the blood sample by tryptic soy agar. Further bacteriological tests showed that the results of CO_2_ requirement, H_2_S production and phage lysis tests were negative, while hydrolysis of urea, growth on thionin and agglutination in sera R were positive. The biotyping characteristics of the strain (CD3) were in accord with that of *B. canis* RM6/66 (Table 2).

### 3.2. Polymerase Chain Reaction Tests

**Figure 1:**
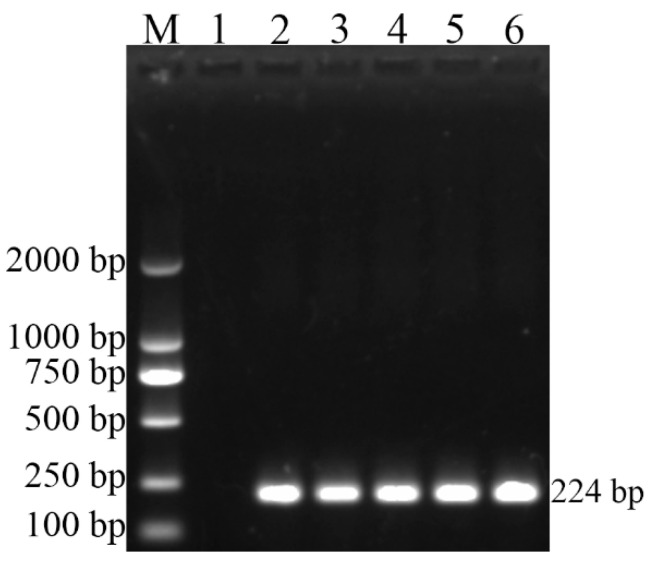
BCSP31-PCR results of the four reference strains and the isolate in this study (CD3). Lane M, molecular weight marker; Lane 1, negative control with no DNA added; Lane 2, DNA extracted from *B. abortus* (544A); Lane 3, DNA extracted from *B. melitensis* (16M); Lane 4, DNA extracted from *B. suis* (S2); Lane 5, DNA extracted from *B. canis* (RM6/66); Lane 6, DNA extracted from the isolated strain (CD3).

**Figure 2:**
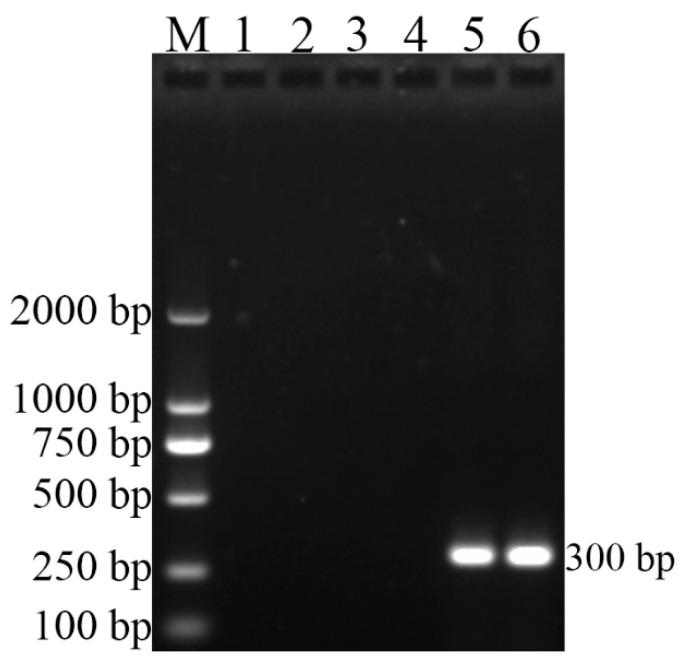
BcSS-PCR results of the four reference strains and the isolate in this study (CD3). Lane M, molecular weight marker; Lane 1, negative control with no DNA added; Lane 2, DNA extracted from *B. abortus* (544A); Lane 3, DNA extracted from *B. melitensis* (16M); Lane 4, DNA extracted from *B. suis* (S2); Lane 5, DNA extracted from *B. canis* (RM6/66); Lane 6, DNA extracted from the isolated strain (CD3).

As Figure 1 shows, the *Brucella* genus-specific PCR (BCSP31-PCR), using all five strains (544A, 16M, S2, RM6/66, and CD3), successfully produced 224-bp PCR amplicon, indicating that CD3 strain belongs to the genus Brucella. Further *B. canis* species-specific PCR (BcSS-PCR) amplified a specific 300-bp amplicon (Figure 2), suggesting that the CD3 isolate is *B. canis*. All amplification results of PCR are listed in Table 3.

### 3.3. MLST Genotyping

Details of allelic profile of each *Brucella* strain are listed in Table 4. The MLST results showed that the isolated *B. canis* strain (CD3) and two *B. canis* strains stored in our lab (WJ5 and YA4) have the same allelic profile, and all three *B. canis* strains from pet dogs in Sichuan belong to genotype ST21.

**Figure 3:**
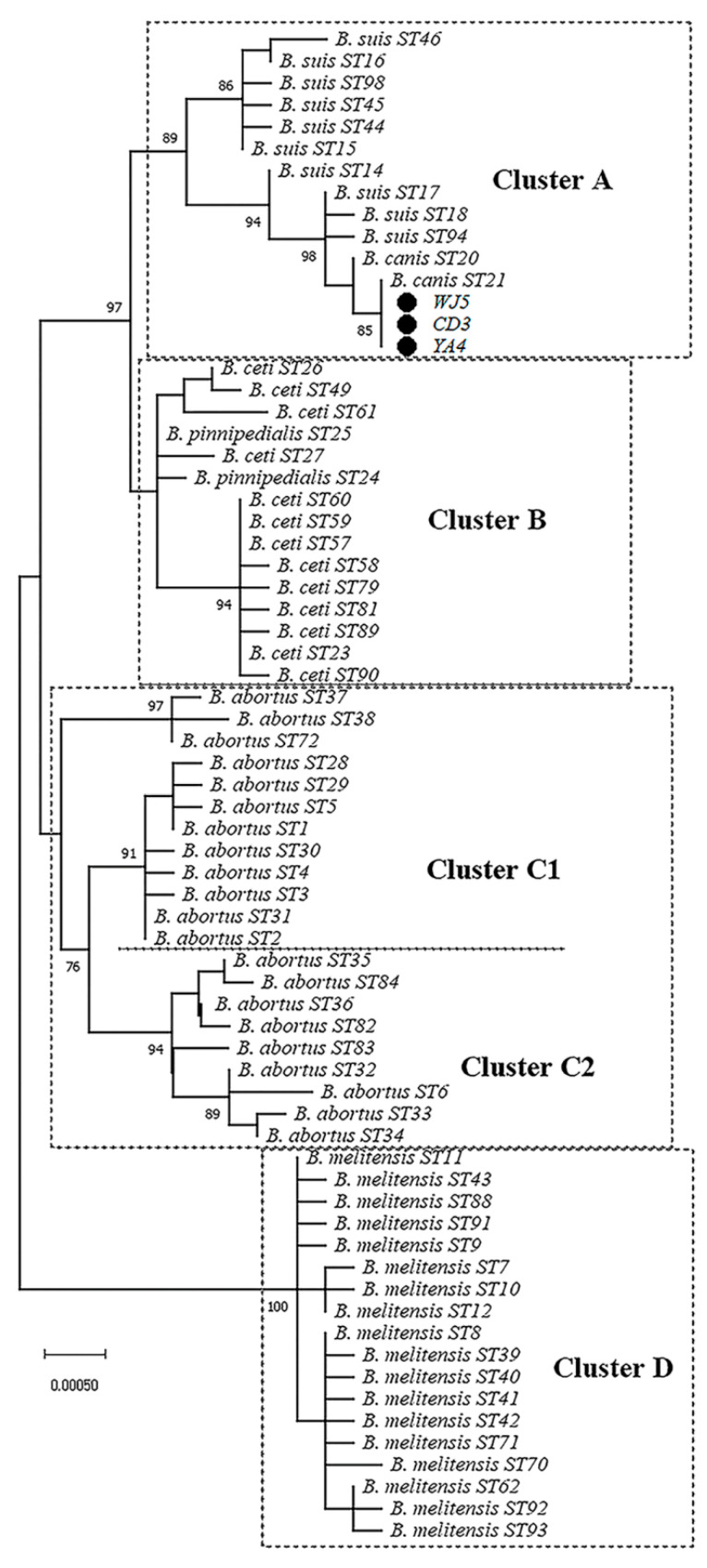
Dendrogram based on the MLST genotyping assay showing relationship of 3 *B. canis* isolates and 66 *Brucella* isolates in MLST database. ● The *B. canis* strains from pet dogs in Sichuan, China.

In order to better understand the genetic relationship of the three *B. canis* strains (CD3, WJ5 and YA4) isolated from pet dogs, sequences of the nine loci were concatenated to conduct phylogenetic analysis with 66 Brucella isolates in the MLST database. As the dendrogram shows (Figure 3), four major clusters (A–D) were formed. The three *B. canis* strains (CD3, WJ5 and YA4) were clustered together with *B. canis* and *B. suis* strains. *B. ceti* and *B. pinnipedialis* were all grouped into cluster B together. *B. abortus* were grouped into cluster C, which split into two subclusters (cluster C1 and C2). *B. melitensis* were clustered into cluster D.

## 4. Discussion

In this study, we diagnosed a pet dog without clinical symptoms as *B. canis* infection in Sichuan Province, China, and a *B. canis* strain (CD3) was successfully isolated. Canine brucellosis is a zoonotic disease that has been underestimated worldwide, posing a public health risk to dogs and humans [3,13,23,24]. In China, serological data on brucellosis in pet dogs are relatively limited. An epidemiological study on brucellosis among 415 pet dogs between 2006–2007 in Beijing showed the seroprevalence was 0.24% (1/415) [25]. In 2012–2013, another study for pet dogs showed the incidence rate was 47.37% (18/38) [26]. These data reveal that the seroprevalence of brucellosis in pet dogs in China has increased in recent years. *B. canis* strains have been identified from dogs after the first isolation in 1984 [4]. Reported infected pet dogs usually showed clinical symptoms, including abortion, low fever, enlarged lymph nodes, obviously enlarged testicle and undulating fever [5,26,27]. In the present study, a pet dog without any clinical symptoms was diagnosed as subclinical infection with *B. canis*. The dog showed healthy on physical examination, while *B. canis* infection was revealed by serological screening and confirmed by subsequent bacteriological and molecular biological tests. This hidden case of *B. canis* infection in pet dog was reported in China for the first time, which will raise public health concerns about contracting *B. canis*. According to other reports, *B. canis* can be transferred from pet dogs to humans through direct contact with infected dogs or their blood or reproductive samples (aborted material, seminal fluid, vaginal discharge, and so forth), and even the spayed or neutered dogs can still shed bacteria in their secretions and urine [3,23]. However, due to the lack of indication of corresponding clinical symptoms, subclinically infected pet dogs are not easy to be detected by owners or veterinarians and may become a risky source of human and animal brucellosis. Transmission of *B. canis* from asymptomatically infected pet dog to human has been reported previously [8]. In New York, an eight-week-old male Yorkshire Terrier, which appeared healthy upon physical exam, was considered as the source of a three-year-old child’s *B. canis* infection because of the genetic similarity between *B. canis* isolates from the child and the pet dog [8]. In addition, human cases of *B. canis* infection have been reported in many countries, including China, the United States, Argentina, and Japan [6,7,8,9,10,11,12]. Considering the convenience and covertness of transmission of *B. canis* from pet dogs to humans, we suggest incorporating *B. canis* screening into the routine medical examination of pet dogs. Reproductive failure (including infertility and abortion) is the main problem in dogs infected with *B. canis* [3]. Reproductive ability of the pet dog in this study has not been tested and needs to be explored.

MLST and phylogenetic analysis was further performed to understand the molecular characteristics and genetic relationship of the three *B. canis* strains (CD3, WJ5 and YA4) isolated from pet dogs in Sichuan Province. Our results showed that the allelic profile of the three *B. canis* strains were identical to each other and all three *B. canis* strains were identified as existing sequence type ST21, which has been identified in *B. canis* strains in Guangxi, China [18], and the United States, Africa, and Europe [15,28]. All three *B. canis* isolates (CD3, WJ5 and YA4) were clustered together with other *B. canis* strains and *B. suis* strains. The result indicates the close relationship between *B. canis* and *B. suis*, which is consistent with findings of Whatmore et al. [15]. Meanwhile, STs of *B. abortus* and *B. melitensis* were grouped into individual clusters, respectively, and strains from marine mammals (*B. ceti* and *B. pinnipedialis*) were grouped into a cluster together. These findings show high resolution of MLST method for phylogenetic genotyping of *Brucella*. In addition, the three *B. canis* strains in our study were isolated in 2015, 2016 and 2019, respectively, and they were from three different cities in Sichuan Province (Wenjiang, Yaan and Chengdu). Therefore, we speculated that ST21 may be the dominant genotype of *B. canis* infecting pet dogs in Sichuan Province in recent years. However, due to the small number of cases, surveys of *B. canis* infection on a larger scale in epidemic areas are needed to better understand the genotype of *B. canis*.

## 5. Conclusions

We first isolated a *B. canis* strain from a subclinically infected pet dog in China, indicating a potential threat to public health posed by subclinical infections in pet dogs. We suggest that screening for *B. canis* should be incorporated into routine medical examination of pet dogs and other companion animals in areas with a history of animal or human brucellosis.

## Figures and Tables

**Table 1:** Primers used in PCR tests and MLST genotyping.

Primer	Sequence (5′-3′)	Amplicon Size (bp)
PCR tests		
BCSP31	F:TGGCTCGGTTGCCAATATCAA	224
	R:CGCGCTTGCCTTTCAGGTCTG	
BcSS	F:CCAGATAGACCTCTCTGGA	300
	R:TGGCCTTTTCTGATCTGTTCTT	
MLST genotyping		
gap	F:YGCCAAGCGCGTCATCGT	589
	R:GCGGYTGGAGAAGCCCCA	
aroA	F:GACCATCGACGTGCCGGG	565
	R:YCATCAKGCCCATGAATTC	
glk	F:TATGGAAMAGATCGGCGG	475
	R:GGGCCTTGTCCTCGAAGG	
dnaK	F:CGTCTGGTCGAATATCTGG	470
	R:GCGTTTCAATGCCGAGCGA	
gyrB	F:ATGATTTCATCCGATCAGGT	469
	R:CTGTGCCGTTGCATTGTC	
trpE	F:GCGCGCMTGGTATGGCG	486
	R:CKCSCCGCCATAGGCTTC	
cobQ	F:GCGGGTTTCAAATGCTTGGA	422
	R:GGCGTCAATCATGCCAGC	
int_hyp	F:CAACTACTCTGTTGACCCGA	430
	R:GCAGCATCATAGCGACGGA	
omp25	F:ATGCGCACTCTTAAGTCTC	490
	R:GCCSAGGATGTTGTCCGT	

**Table 2:** Biotyping characteristics of *B. canis* RM6/66 and the isolate CD3 from the subclinically infected pet dog in Sichuan, China.

Isolate	CO_2_ Requirement	H_2_S Production	Hydrolysis of Urea	Agglutination in Sera			Growth on Dyes		Phage Lysis Tests		
				A	M	R	Thionin	Basic Fuchsin	Tb	BK_2_	Wb
CD3	- ^1^	-	+ ^2^	-	-	+	+	-	-	-	-
RM6/66	-	-	+	-	-	+	+	-	-	-	-

^1^ negative result. ^2^ positive result.

**Table 3:** Results of two PCR assays of all five strains used in this study.

Species	Strains	PCR Results	
		BCSP31-PCR	BcSS-PCR
*B. abortus*	544A	+ ^1^	- ^2^
*B. melitensis*	16M	+	-
*B. suis*	S2	+	-
*B. canis*	RM6/66	+	+
*B. canis*	CD3	+	+

^1^ amplicon by PCR. ^2^ no amplicon by PCR.

**Table 4:** Allelic profiles of three *B. canis* strains isolated from pet dogs in Sichuan, China.

Strains	Host	Year	Gap	aroA	glk	dnaK	gyrB	trpE	cobQ	int_hyp	omp25	ST
WJ5	dog (golden retriever)	2015	1	6	4	1	5	3	5	4	5	21
YA4	dog (poodle)	2016	1	6	4	1	5	3	5	4	5	21
CD3	dog (golden retriever)	2019	1	6	4	1	5	3	5	4	5	21

## Data Availability

Publicly available datasets were analyzed in this study. These data can be found here: https://pubmlst.org/Brucella, accessed on 9 August 2021.
